# A giant left ventricular pseudoaneurysm observed using multiple imaging modalities

**DOI:** 10.1093/ehjcr/ytae025

**Published:** 2024-01-17

**Authors:** Nobuaki Kobayashi, Yasuhiro Kawase, Masamichi Takano, Masahiro Fujii, Kuniya Asai

**Affiliations:** Department of Cardiovascular Medicine, Nippon Medical School Chiba Hokusoh Hospital, 1715 Kamagari, Inzai, 270-1694 Chiba, Japan; Department of Cardiovascular Surgery, Nippon Medical School Chiba Hokusoh Hospital, Chiba, Japan; Department of Cardiovascular Medicine, Nippon Medical School Chiba Hokusoh Hospital, 1715 Kamagari, Inzai, 270-1694 Chiba, Japan; Department of Cardiovascular Surgery, Nippon Medical School Chiba Hokusoh Hospital, Chiba, Japan; Department of Cardiovascular Medicine, Nippon Medical School, Tokyo, Japan

A 65-year-old man with a history of angina pectoris seven years prior and inferior ST-segment elevation myocardial infarction (STEMI) three years prior presented to our hospital due to tissue protrusion at the inferior end of sternum. Seven years ago, he received stents implantation in posterior descending artery of dominant left circumflex artery at another hospital. Three years ago (i.e. four years after the first procedure), he was transferred to our hospital with inferior STEMI. Emergency coronary angiography showed old stents occlusion (white arrow in *Panel A* and Supplementary material online, *Video S1*). Due to difficulty in visualizing the occluded proximal end of the distal coronary artery lesion, reperfusion therapy was not administered. Three days after the STEMI onset, he suffered ventricular septal perforation and ventricular free wall rupture and underwent surgical patch repair. Three years after the ventricular rupture, he was readmitted to our institution with tissue protrusion at the inferior end of the sternum. Computer tomography (CT) was used to evaluate the protruding mass (asterisks in sagittal plane CT image; *Panel B*) and revealed contrast medium pooling (11 cm × 12 cm × 6 cm) inferior to the patch repair site of the left ventricle (LV) (yellow arrow in coronal plane CT image; *Panel C*). The pooling was diagnosed as a left ventricular pseudoaneurysm (PSA) by echocardiography (*Panel D* and Supplementary material online, *Video S2*), four-dimensional magnetic resonance imaging (red and green arrows showing fast and slow blood flow, respectively, in *Panel E* and Supplementary material online, *Video S3*), and left ventriculography (*Panel F* and Supplementary material online, *Video S4*), with findings showing blood flow from the LV through a narrow hole (white arrow heads) to the PSA. Re-surgical patch repair was performed successfully. The PSA was extremely fragile, and the PSA easily ruptured once the procedure started after cardiopulmonary pump on.

**Figure:**
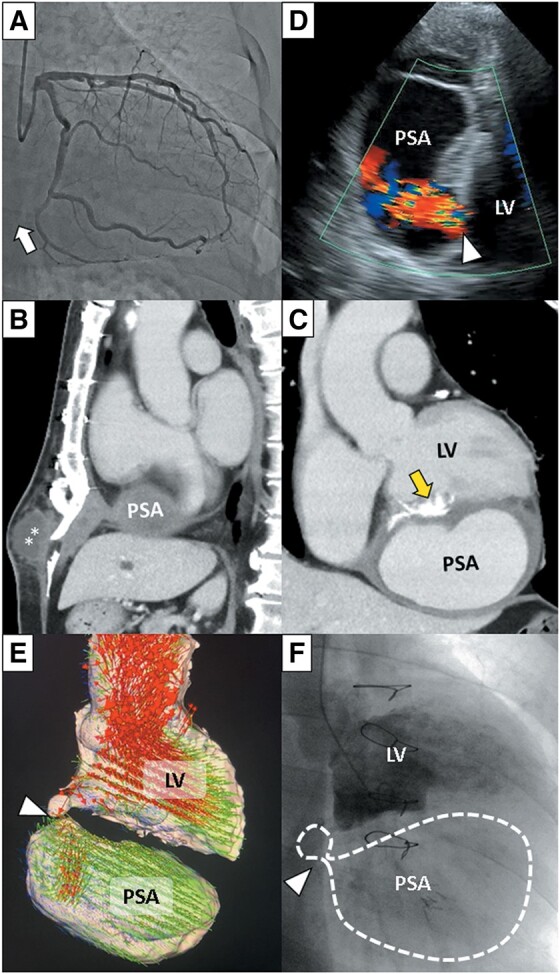


## Supplementary material

Supplementary material is available at *European Heart Journal – Case Reports* online.

**Consent**: Informed consent has been obtained by the subject for the publication. The authors confirm written consent for the submission and publication of this case report.

**Funding**: None declared.

## Data availability

No data were generated or analysed during the study.

## Supplementary Material

ytae025_Supplementary_Data

